# Slips of Action and Sequential Decisions: A Cross-Validation Study of Tasks Assessing Habitual and Goal-Directed Action Control

**DOI:** 10.3389/fnbeh.2016.00234

**Published:** 2016-12-20

**Authors:** Zsuzsika Sjoerds, Anja Dietrich, Lorenz Deserno, Sanne de Wit, Arno Villringer, Hans-Jochen Heinze, Florian Schlagenhauf, Annette Horstmann

**Affiliations:** ^1^Department of Neurology, Max-Planck Institute for Human Cognitive and Brain SciencesLeipzig, Germany; ^2^Department of Psychiatry and Psychotherapy, Campus Charité Mitte, Charité – Universitätsmedizin BerlinBerlin, Germany; ^3^Department of Neurology, Otto-von-Guericke UniversityMagdeburg, Germany; ^4^Department of Clinical Psychology, University of AmsterdamAmsterdam, Netherlands; ^5^Amsterdam Brain and Cognition, University of AmsterdamAmsterdam, Netherlands; ^6^Mind and Brain Institute, Charité and Humboldt UniversityBerlin, Germany; ^7^Department of Behavioral Neurology, Leibniz Institute for NeurobiologyMagdeburg, Germany; ^8^Integrated Research and Treatment Center Adiposity Diseases, Leipzig University Medical CenterLeipzig, Germany

**Keywords:** goal-directed, habit, model-based, model-free, cross-validation, sequential decision making, slips-of-action, reinforcement learning

## Abstract

Instrumental learning and decision-making rely on two parallel systems: a goal-directed and a habitual system. In the past decade, several paradigms have been developed to study these systems in animals and humans by means of e.g., overtraining, devaluation procedures and sequential decision-making. These different paradigms are thought to measure the same constructs, but cross-validation has rarely been investigated. In this study we compared two widely used paradigms that assess aspects of goal-directed and habitual behavior. We correlated parameters from a two-step sequential decision-making task that assesses model-based (MB) and model-free (MF) learning with a slips-of-action paradigm that assesses the ability to suppress cue-triggered, learnt responses when the outcome has been devalued and is therefore no longer desirable. MB control during the two-step task showed a very moderately positive correlation with goal-directed devaluation sensitivity, whereas MF control did not show any associations. Interestingly, parameter estimates of MB and goal-directed behavior in the two tasks were positively correlated with higher-order cognitive measures (e.g., visual short-term memory). These cognitive measures seemed to (at least partly) mediate the association between MB control during sequential decision-making and goal-directed behavior after instructed devaluation. This study provides moderate support for a common framework to describe the propensity towards goal-directed behavior as measured with two frequently used tasks. However, we have to caution that the amount of shared variance between the goal-directed and MB system in both tasks was rather low, suggesting that each task does also pick up distinct aspects of goal-directed behavior. Further investigation of the commonalities and differences between the MF and habit systems as measured with these, and other, tasks is needed. Also, a follow-up cross-validation on the neural systems driving these constructs across different paradigms would promote the definition and operationalization of measures of instrumental learning and decision-making in humans.

## Introduction

Instrumental decision-making requires learning and executing adequate behavior efficiently in relevant situations in order to obtain desired outcomes. Based on an extensive body of animal research, this ability is thought to rely on the functioning of two parallel systems: a reflexive habitual system and a deliberate goal-directed system (Dickinson, 1985; Balleine and Dickinson, 1998a). The habit system is believed to be an evolutionary basal system, suggested to mainly rely on dorsolateral striatal areas (Yin et al., 2004, 2006; Tricomi et al., 2009; Wunderlich et al., 2012a). Habits are “stamped in” by past reinforcements until they are performed in an automatic routine. The habit system is inflexible and suboptimal in changing environments, but it offers the advantage to free up cognitive resources, allowing the allocation of attention to parallel tasks. In contrast, goal-directed behavior has shown to largely involve prefrontal cortical and dorsomedial striatal brain areas (Corbit and Balleine, 2003; Killcross and Coutureau, 2003; Yin et al., 2005a; Valentin et al., 2007; de Wit et al., 2009; but see Jonkman et al., 2009), and is characterized by flexible behavior, which is more easily adaptable in the face of changing contingencies. However, it is thought to be computationally more demanding than the habit system, and the ability to engage the goal-directed system effectively seems to depend on trait factors such as healthy aging (Eppinger et al., 2013; de Wit et al., 2014) and cognitive capacities (Otto et al., 2013; Smittenaar et al., 2013; Schad et al., 2014) or state conditions, including stress (Schwabe and Wolf, 2009; Otto et al., 2013; Radenbach et al., 2015). There is growing evidence that deficient instrumental decision-making based on the dual-systems theory is implicated in multiple disorders (e.g., Gillan et al., 2011; Sjoerds et al., 2013; Horstmann et al., 2015; Voon et al., 2015b; McKim et al., 2016; Reiter et al., 2016). Human behavior, however, might be influenced by a wide variety of unrelated external or internal factors (i.e., social conventions, cultural context or financial situations), rendering it more noisy than e.g., rodent behavior despite the large overlap between human and rodent instrumental systems (Balleine and O’Doherty, 2010). This increases the complexity to measure individual constructs in humans. Together with the need to apply cognitive measurements in pathological samples, this advances the prerequisite to optimize the assessment of these instrumental behaviors.

To adequately assess the degree to which the two proposed systems are used in instrumental choices, it is essential to ensure suitable instruments that objectively assess covert sub-processes contributing to the constructs and that are simultaneously straightforward for intuitive analyses and application in patient samples (Huys et al., 2016). Throughout the past decade, distinct paradigms to study the two systems in humans have been operationalized based on different methodological and historical perspectives of habitual vs. goal-directed behavior (Doll et al., 2012; Dolan and Dayan, 2013). They can be distinguished by whether the paradigm captures the ongoing contingency updating process or largely established behavioral schemata, processes that are not necessarily independent of each other (Gillan et al., 2015). Further variations lie in the focus on central sub-processes underlying habitual and goal-directed choices. For example, goal-directed behavior is complex and involves multiple sub-processes including forward planning, outcome contingency weighting, search processes and abstract inference (Hampton et al., 2006; Abe and Lee, 2011; Daw et al., 2011; Doll et al., 2012). Change in outcome value or contingencies (e.g., outcome devaluation or changes in outcome probabilities) provides the canonical assay of behavioral flexibility as related to the balance between goal-directed vs. habitual control. Frequently used tasks, such as a slips-of-action paradigm (de Wit et al., 2012b) and sequential decision-making paradigm (Daw et al., 2011) assess the ability to rapidly adjust behavior to changes in outcome value.

Classically, the relative involvement of the goal-directed and habitual systems in instrumental choices has been studied in animals by (selective) outcome devaluation procedures (Adams and Dickinson, 1981; Balleine and Dickinson, 1998b), a method that has been adapted to human research (Valentin et al., 2007; Tricomi et al., 2009; Horstmann et al., 2015). Devaluation of an outcome (O) that has been associated with a stimulus (S) will change response (R) behavior when under control of the goal-directed system, as R-O contingencies are represented in the goal-directed system. However, once S-R habitual responding to a stimulus is established, the outcome is no longer taken into account in the choice behavior; therefore, devaluation of the outcome will not immediately influence habitual responding to the stimulus but only after gradual update of the stimulus value after repeated outcome feedback. Following the same line of reasoning, a slips-of-action paradigm was developed (de Wit et al., 2012b), in which participants learn stimulus-reward contingencies. After training, an instructed devaluation phase assesses whether participants can suppress previously learned responses that yield no-longer-valuable outcomes, while continuing to respond for still-valuable outcomes. A failure to do so, as reflected in “slips of action” towards devalued outcomes, is interpreted as relative reliance on S-R habitual—as opposed to goal-directed—control. Crucially, a devaluation sensitivity index (DSI) is calculated based on the difference in responding between these two trial types, providing a single parameter that represents the relative involvement of the habit vs. goal-directed system in action control. This task has been used extensively to study goal-directed and habitual action control in healthy participants (de Wit et al., 2009, 2012b) following dopamine (de Wit et al., 2012a) and serotonin level reductions (Worbe et al., 2015) and in patient samples, including obsessive-compulsive disorder (Gillan et al., 2011), alcohol dependence (Sjoerds et al., 2013), Gille de La Tourette syndrome (Delorme et al., 2016), Parkinson’s Disease (de Wit et al., 2011; O’Callaghan et al., 2013) and autism spectrum disorders (Geurts and de Wit, 2014). It remains unclear, however, how devaluation sensitivity, as assessed with this task, relates to other paradigms assessing goal-directed and habitual control, such as the model-based (MB) and model-free (MF) reinforcement learning (RL) algorithms used during sequential decision making.

RL theory aims to formalize decision-making processes such as goal-directed and habitual learning by describing distinct underlying computational mechanisms. To this end, in addition to analyzing observable behavior such as accuracy, reaction times and win-stay probabilities, generative models are implemented to infer parameters that underlie the observed behavior. One of the frequently used RL models follows the temporal difference (TD) theory (Sutton and Barto, 1998), which is closely linked to habitual learning. It provides a “MF” update rule to learn action values based on past reinforcements. Goal-directed instrumental learning has also been proposed to have a formal counterpart in RL, in a family of algorithms known as “MB” RL (Daw et al., 2005; Rangel et al., 2008; Redish et al., 2008). The MB system uses a model of the environment for flexible forward planning. Resembling the goal-directed system, it contains knowledge on the causal relationship between actions and outcomes. In the context of RL theory, one task to study goal-directed vs. habitual responding with the MB and MF algortihms, respectively is the two-step sequential decision making task (Daw et al., 2011) in combination with computational modeling of decision making using MB and MF algorithms. This two-step task has been extensively used in the past years to study MB vs. MF learning in healthy and diseased samples (Daw et al., 2011; Wunderlich et al., 2012b; Eppinger et al., 2013; Otto et al., 2013; Deserno et al., 2015a,b; Gillan et al., 2015; Radenbach et al., 2015; Voon et al., 2015b; Morris et al., 2016; Reiter et al., 2016; Worbe et al., 2016).

The increasing availability of instruments measuring goal-directed and habitual behavior increases the necessity for cross-validation of different paradigms on the assessment of the two central constructs. Recently, Friedel et al. (2014) have performed a valuable cross-validation study on the goal-directed and habit constructs assessed by a selective devaluation task (Valentin et al., 2007) and the two-step sequential decision-making task (Daw et al., 2011). They found specific cross-correlation between MB choices during sequential decisions and goal-directed behavior after devaluation. This suggests a single framework underlying both task measures, speaking in favor of construct validity of both measurement approaches. However, further comparable research on cross-validation of instrumental decision-making between other tasks is needed. Another recent study directly manipulated MB learning with habitual responding within one paradigm: they used an adjusted two-step sequential decision-making task, including a later phase that provided a DSI (Gillan et al., 2015). By using a median split on this DSI, they defined groups of participants using predominantly goal-directed or habitual responding. They found that MB control during the first phase of the task protected from established habitual responding during the last phase measured by devaluation sensitivity. This further indicates an overlap between MB learning and established goal-directed behavior.

We would like to extend this line of research by cross-validating MB control and goal-directed responding between two different tasks that have been most commonly used in the recent body of literature. We will correlate parameters describing MB and MF control from the two-step sequential decision-making task (Daw et al., 2011) with a DSI from the slips-of-action paradigm (de Wit et al., 2012b; Worbe et al., 2015), which measures the relative balance of goal-directed and habitual choices on a gradual scale. We hypothesize a positive association between the measure of MB behavior and the DSI. In other words, participants who show more MB behavior in the two-step task are expected to be better able to respond selectively for still-valuable outcomes, while suppressing slips of action towards no-longer valuable outcomes in the slips-of-action paradigm. We additionally explore a possible association between the tasks on the habit system, expecting a negative correlation between MF behavior and the DSI. We furthermore assess the role of higher-order cognitive capacities, measured by widely used neuropsychological tests, in the recruitment of the goal-directed/MB and habitual/MF systems.

## Materials and Methods

### Participants

A total of 28 healthy participants (12 females, mean age: 27, see Table 1) performed both paradigms. Based on the previous cross-validation study by Friedel et al. (2014), showing effects sizes between 0.5 and 0.7, an a-priori power analysis (G*Power version 3.1.9.2) showed that for the current study a sufficient sample size would lie between *N* = 13 and *N* = 34. Volunteers were highly educated non-smokers without indication for major depression as measured with the Beck’s Depression Inventory (BDI), cut-off value 18 (Beck et al., 1996). Non-verbal intelligence was assessed with the Wiener Matrizen Test (WMT, The Viennese Matrices Test; Formann and Piswanger, 1979). Visual short-term memory was tested with a Visual Association Test (VAT), a computerized version of the Visual Paired Association (VPA) Test, part of the Wechsler Memory Scale (Wechsler, 1987, 2006). In this test participants have to memorize the combination of shape and color of six different stimulus pairs. Cognitive speed was assessed with the digit-symbol-substitution test (DSST; Wechsler, 1955). These cognitive measures were included to examine their potential relation to performance on each task.

**Table 1:** **Sample descriptives**.

Descriptive	Mean	SD	Range
Age	27.04	3.415	21–34
Gender (female): *N*, %	*N* = 12	42.90%	
Beck depression inventory (BDI)	3.68	3.422	0–14
Wiener matrizen test (WMT)
Score	18.96	3.854	11–24
IQ	120.38	12.249	95–136.5
Visual association test (VAT)	12.11	3.891	3–18
Digit symbol substitution test (DSST)	87.05	10.741	60–110

Participants were recruited from the database at the Max Planck Institute for Human Cognitive and Brain Sciences in Leipzig, Germany. All participants were financially compensated for participation with €7-per hour in addition to a monetary reward acquired during the experimental tasks. The study was approved by the Ethics Committee of the University of Leipzig, Germany, and conducted in accordance with the Declaration of Helsinki. Written informed consent was obtained from all participants prior to the study.

### Paradigms

#### Three-Phase Instrumental Learning Task

**Figure 1:**
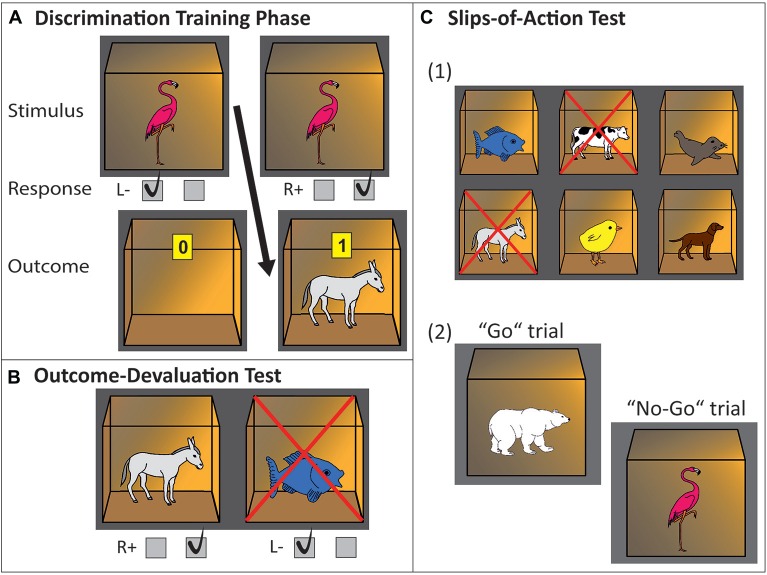
**Experimental paradigm, three-phase instrumental learning task. (A)** Discrimination training phase. In this example, a flamingo stimulus printed on the front of a closed box indicates that pressing the right key will open the box and will be rewarded with a donkey and points inside of the box. Pressing the left key will not be rewarded (empty open box is revealed). **(B)** Outcome-devaluation test. In this example, two open boxes are presented with a donkey and fish inside. The cross superimposed on the fish signals this outcome is no longer worth any points. The accurate response in this example would be pressing the right key (which yielded the still-valuable donkey outcome during the learning phase). **(C)** Slips-of-action test. **(1)** Participants are first presented with the six outcomes. In this example, donkey and cow are superimposed with a cross, indicating that the response leading to these outcomes will now result in subtraction of points (devaluation). The other animal outcomes are still valuable. **(2)** Afterwards, in rapid succession animal stimuli are presented on the outside of the boxes. Participants are instructed to press the correct key if a stimulus indicates the availability of a still-valuable outcome inside the box (“Go”, example: polar bear stimulus signaling fish outcome), but withhold responding if the outcome inside the box has been devalued (“No-Go”, example: flamingo stimulus signaling donkey outcome).

A simplified version of the slips-of-action paradigm, an instrumental learning task developed by de Wit et al. (2012b) was used, which has been successfully applied in previous studies (e.g., Worbe et al., 2015; Delorme et al., 2016). In the current study, pictures of animals instead of fruit pictures were used. The task consists of three phases, a discrimination training phase to learn S-R-O associations and an outcome devaluation phase and slips-of-action phase to test for the strength of learned S-R-O associations (see Figure 1). The slips-of-action phase provides a DSI (for detailed explanation, see below), which encompasses a “balance” measure of relative goal-directed and habitual control. We do report results on the other phases of the task, as is done in all previous studies using the same task (e.g., de Wit et al., 2011, 2014; Geurts and de Wit, 2014; Worbe et al., 2015; Delorme et al., 2016). However, the current study solely aims to assess the parallels between different tasks in measuring relative involvement of goal-directed/MB and habitual/MF control. Therefore the DSI of this task is of main interest for correlational analyses to the current study.

##### Discrimination training phase

During the first phase, the discrimination training phase, participants learned by trial-and-error to respond (R) with a left or right button press to stimuli (S) in order to gain outcomes (O) that are worth points representing monetary reward. Participants were instructed to earn as many points as possible. A trial started with a box displayed in the middle of the screen, with a picture of an animal printed on the front side. Participants were instructed that the box could be opened with either a left or right button press, but that only one of the two buttons is the correct one, rendering another animal plus a monetary reward in the opened box. When pressing the incorrect button, the box would open, but it would be left empty, without a monetary reward (zero points won). Six different possible stimuli, displayed in a randomized order over the trials, would lead deterministically (i.e., with a 100% contingency) to six different outcomes in the case of a correct response. For three stimuli a right button press would lead to the outcome and a monetary reward, whereas for the other three a left button press would be the correct one to obtain an outcome plus monetary reward. This phase comprised eight blocks and a total of 96 trials. Dividing the task into blocks with randomized stimulus order within each block aided in measuring a learning effect across blocks, and ensured that participants learned all stimuli evenly divided throughout the experiment, instead of randomly seeing only a high amount of repetitions of one stimulus e.g., at the end of the training. Each stimulus was displayed 16 times in order for all participants to adequately learn the S-R-O associations.

##### Outcome-devaluation test phase

Following the discrimination training phase, an outcome-devaluation test phase assessed the strength of goal-directed R-O associations learned during the training phase. Here, outcomes (again, open boxes with animal icons) were displayed in pairs; one that was previously associated with a left response and one with a right response. In each trial, one of the outcomes was devalued (i.e., it would no longer produce a monetary reward), indicated by a red cross superimposed on the devalued outcome. Now, participants had to use their knowledge of the R-O relationships to (re)direct their choices towards the still-valuable outcome, by pressing the button that had lead to this outcome during the discrimination training phase. This phase was comprised of 36 trials. Participants were not directly given feedback on each trial, but instead were instructed that correct button presses would still earn them points and that they would be shown their total score at the end of the test phase.

During the discrimination training phase and the outcome-devaluation test phase, we assessed the total percentage correct responses (task accuracy). Due to non-normal distribution of all outcome measures (including accuracy in the learning phase), non-parametric testing was used. The Friedman test was applied to test for performance differences across the eight equal blocks of the training-phase, to check for instrumental learning effects over time.

##### Slips-of-action phase

During the slips-of-action phase, the balance between goal-directed and habitual learning systems was directly assessed and hence, this phase is of main interest for cross-validation with the sequential decision-making task (see below).

This phase was comprised of nine blocks, with a total of 108 trials. At the beginning of each block an instruction screen with six possible outcomes (open boxes with animal icons inside) was shown for 5 s, two of them superimposed with a cross. The cross indicated devaluation of those outcomes, and that responding to the stimulus associated with those outcomes would consequentially no longer earn points. After this screen, stimulus pictures were shown in rapid succession. Participants had to respond as fast as possible with a correct button-press to stimuli (closed boxes with an animal icon printed on the front) associated with still-valuable outcomes, and withhold their response for stimuli associated with devalued outcomes. Each stimulus remained on the screen for a fixed 1000 ms, during which the participant had to respond or withhold their response, respectively. The next trial started after an inter-trial interval (ITI) of 1000 ms. As in the outcome-devaluation phase, also in this phase no direct feedback was given, in order to prevent new learning. Instead, the total amount of points was shown at the end of the phase. During each block, each of the six stimuli was shown twice in semi-random order, with the exception that stimuli were never directly repeated. Throughout the nine blocks, each outcome was devalued three times, resulting in 36 trials where the outcome was devalued, and 72 trials with still valuable outcomes.

In this phase, response tendencies through direct S–R associations (related to the habit system) should lead to commission errors on trials showing stimuli associated with the devalued outcomes. Contrarily, successful selective inhibition on the basis of outcome value should be suggestive of dominant goal-directed control through more complex S-R-O associations, which is mediated by anticipation and evaluation of the consequent outcome (see e.g., Gillan et al., 2011; de Wit et al., 2012a,b). We calculated the DSI for the slips-of-action phase by subtracting percentages of responses made toward devalued outcomes from percentages of responses made toward still valuable outcomes, according to the following formula: ((*N* valued responses/*N* total responses) − (*N* devalued responses/*N* total responses)). Following the explanation above, this DSI during the slips-of-action phase is a “balance” measure of relative goal-directed and habitual control, and hence of main interest for the cross validation with the sequential decision-making task.

##### Baseline test phase

As a control test for general inhibitory impairments, participants also performed a baseline test of inhibitory control. This test closely resembled the slips-of-action phase, except that the decision to respond or withhold could be based directly on stimulus identity as opposed to outcome anticipation. To this end, at the beginning of each block a screen with the six possible stimuli (closed boxes with animal printed on the outside) was shown, two of them superimposed with a cross. This time participants simply had to withhold their responses for the stimuli that had been superimposed with a cross (“stimulus devaluation”). Again, no direct feedback was given, but they were shown the total amount of earned points at the end of the phase. Importantly, the baseline test controls for outcome-based responding, as it is independent of the outcomes. However, it does not control for S-R associated behavior, thus the test might also be driven by strong S-R associations, which could be indicative of habitual behavior. The order of slips-of-action and baseline test phase was counterbalanced across participants.

#### Two-Step Sequential Decision-Making Task

**Figure 2:**
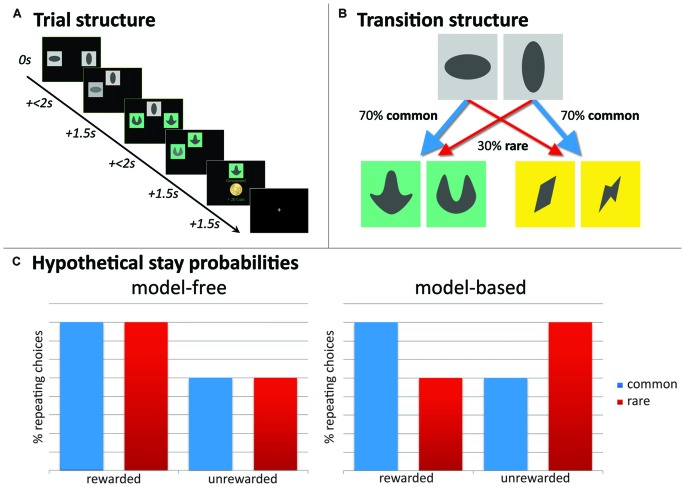
**Experimental paradigm, two-step sequential decision-making task. (A)** Example of a trial-sequence with timing; **(B)** state-transition probabilities indicating common and rare transitions; **(C)** hypothetical full model-free (MF) and full model-based (MB) choice strategies would result in these choice patterns. Depicted here, stay probability plots for first step choices as a function of reward (reward vs. no reward) and state (common vs. rare). A main effect of reward guides MF choice strategies, whereas MB choice strategies show a reward * state interaction.

A two-step Markov sequential decision-making task (Daw et al., 2011; see Figure 2) was used to assess the degree of MB and MF behavioral control. The applied version was identical to previous work from our group (Friedel et al., 2014; Sebold et al., 2014; Deserno et al., 2015a,b; Reiter et al., 2016). The task consisted of 201 two-stage trials. Within each trial, participants made two (stage 1, stage 2) sequential choices out of two stimuli to finally receive a monetary reward after the second stage (Figure 2A). At the first stage, participants selected within 2 s one of two stimuli displayed in gray boxes. Responses slower than 2 s were invalid. The chosen stimulus moved to the top middle of the screen and remained displayed during the second stage, while the non-chosen stimulus faded after the choice was made. At the second stage, participants again chose between two stimuli in differently colored pairs of boxes. The position of the stimuli on the left or right side of the screen in both stages was randomized and participants were explicitly instructed that the position of the stimuli was not relevant. The second-stage choice could either be rewarded with 20 eurocents (displaying a coin), or was not rewarded (displaying a zero). Feedback (reward or no reward) after the second choice was delivered in a probabilistic manner following slowly changing Gaussian random walks (see Daw et al., 2011). Participants trained for 55 trials before performing the actual task and were explicitly introduced to the task-structure, similar to Daw et al. (2011). They were informed that they would get financial reimbursement after the task with an amount depending on the total reward received during the task.

##### Stay probabilities

Crucial to this task is that presentation of second-stage pairs depends probabilistically on first-stage choices: each of the first-stage choices was predominantly associated with one of the two second-stage stimulus pairs (70% → common state) and less with the other (30% → rare state; see Figure 2B). These state transition probabilities were fixed during the experiment. A MF agent disregards these transition probabilities and stays with the first-stage actions that have led to a reward after a second-stage choice. This indicates a main effect of reward on stay probabilities at the first stage: the probability that the same action will be repeated in the subsequent trial. Contrary, a MB agent does take into account these transition probabilities and accordingly, contains a “model” of the task. In other words, the MB system increases the chance to switch at the first-stage after a reward was delivered following a “rare” transition, but increases stay behavior at the first-stage after receiving no reward after a “rare” transition. This indicates a reward-by-state interaction effect on stay probabilities. See Figure 2C for a hypothetical representation of stay probabilities for a pure MF and pure MB learner, respectively. For task descriptive analyses, individual stay probabilities, as stay-switch behavior was defined as a function of reward (reward vs. no reward) and state (common vs. rare), are subjected to a repeated-measures analysis of variance (ANOVA) with reward and state as within-subjects factors.

##### Computational modeling

We used computational modeling in the analyses of choice behavior, to deduce covert control strategies in solving the task, based on the MF or MB system. In line with Daw et al. (2011), we used RL models that learn choice values (Q) through prediction errors. To this end we distinguish the three pairs of stimuli in the two stages (first stage: *S*_A_, second stage: *S*_B_, *S*_C_), which are followed by an action *a*. First, trial-by-trial MF (*Q*_MF_) stimulus values were calculated with a State-Act-Reward-State-Act (SARSA) (λ) model as follows:

(1) $$Q_{\text{MF}}(S_{\text{i},\text{t+1}},a_{\text{i},\text{t+1}})\;=\;Q_{\text{MF}}(S_{\text{i},\text{t}},a_{\text{i},\text{t}})+\alpha_{i}\delta_{\text{i},\text{t}}$$

Here i denotes the stage (first-stage: i = 1; second stage: i = 2), and t denotes the trial. In equation 1, δ refers to the trial-by-trial prediction error used to update the stimulus value, weighted by learning rate α. The prediction error is computed as the difference between expected value and obtained reward (*r*):

(2) $$\delta_{\text{i},\text{t}}\;=\;r_{\text{i},\text{t}}+Q_{\text{MF}}(S_{\text{i+1},\text{t}},a_{\text{i+1},\text{t}})-Q_{\text{MF}}(S_{\text{i},\text{t}},a_{\text{i},\text{t}})$$

Note that *r*_1,t_ = 0 because no reward is delivered after a first-stage choice, and Q_MF_ (*S*_3,t_, *α*_3,t_) = 0 because the task only has two states. First-stage values are additionally updated by a stage-skipping parameter λ, which connects the two stages and allows the reward prediction error at the second stage to modulate first-stage values:

(3) $$Q_{\text{MF}}(S_{\text{1},\text{t+1}},a_{\text{1},\text{t+1}})\;=\;Q_{\text{MF}}(S_{\text{1},\text{t}},a_{\text{1},\text{t}})+\alpha_{1}\lambda \delta_{\text{2},\text{t}}$$

Next, the MB algorithm learns values by forward planning, and computes first-stage values by merely multiplying the better option at the second stage with the transition probabilities:

(4) $$\begin{array}{cc} Q_{MB}(S_{\text{A}},a_{\text{j}})\;=\; & P(S_{\text{B}}\text{|}S_{\text{A}},a_{\text{j}})maxQ_{\text{MF}}(S_{\text{B}},a)\\ & +P(S_{\text{C}}\text{|}S_{\text{A}},a_{\text{j}})maxQ_{\text{MF}}(S_{\text{C}},a) \end{array}$$

This simplified approach to MB control is justified because participants are extensively trained on the transition probabilities (also shown in Daw et al., 2011). Finally, these MF and MB decision-values are connected in a hybrid algorithm:

(5) $$Q(S_{\text{A}},a_{\text{j}})\;=\;\omega Q_{\text{MB}}(S_{\text{A}},a_{\text{j}})+(1-\omega )Q_{\text{MF}}(S_{\text{A}},a_{\text{j}})$$

In this equation ω is a free weighting parameter, which connects the MB and MF values. Therefore, ω represents the relative influence of the MB and MF system that is, other than two separate parameters describing MB and MF choices (see below), a parameter of interest in the correlation with the goal-directed/habit parameters from the slips of action task.

Finally, to connect the calculated values to choices, we used an observation model following the softmax choice rule. This softmax observation model transforms the obtained values into choice probabilities with three parameters: the free inverse temperature parameter (*β*_i_) shows deterministic choices and is allowed to differ between the two stages (*β*_1_ and *β*_2_) and a repetition parameter (ρ) accounting for perseveration of first-stage choices:

(6) $$p\left(a_{\text{i},\text{t}}\;=\;a\text{|}S_{\text{i},\text{t}}\right)\;=\;\frac{\text{exp}\left(\beta_{\text{i}}\left[Q(S_{\text{i},\text{t}},a)+\rho \;*\;rep(a)\right]\right)}{\sum_{a^{\prime }}\text{exp}\left(\beta_{\text{i}}\left[Q(S_{\text{i},\text{t}},a^{\prime })+\rho \;*\;rep(a^{\prime })\right]\right)}$$

To connect the choices to the values of the MB and MF system individually, we calculated separate free inverse temperatures for the two systems (*β*_MB_ and *β*_MF_) that specify the degree to which action choices follow from the MB and MF action values respectively. To this end we multiplied the first-stage stochasticity parameter *β* with ω: *β*_MB_ = ω * *β* and *β*_MF_ = (1 − *ω*) * *β* (see Otto et al., 2013). These two parameters facilitate examination of individual differences in the influence of either the MB or MF system and are therefore used in the correlation analyses with the slips-of-action task.

Bounded parameters were fitted by transformation to a logistic (α, λ, ω) or exponential (*β*) distribution in order to obtain normally distributed parameter estimates. To infer the maximum-a-posteriori estimate of each parameter for each subject, we set the prior distribution to the maximum-likelihood estimates given the data of all participants, and subsequently used Expectation-Maximization (Huys et al., 2011, 2012).

#### Correlation Between the Two Paradigms

We were interested whether MB and MF updating was associated with goal-directed/habitual choices. Therefore, the *β*_MB_ and *β*_MF_ parameters from the two-step task, describing MB and MF choice behavior respectively, were correlated with the DSI of the slips-of-action phase. The DSI parameter indicates the balance of goal-directed and habitual behavior, and was computed by calculating the difference between percentages of responses made toward valuable outcomes minus percentages of responses made toward devalued outcomes. We expected a positive association between *β*_MB_ and the DSI, and a negative association between *β*_MF_ and the DSI. If a strong association of both *β*_MB_ and *β*_MF_ with DSI could be found, this could reflect a positive association between the DSI and the balance score of the two-step task, the weighting parameter ω, computed using the modeling approach as described above. Therefore, we also performed a confirmatory correlation analysis between these “balance” parameters of the two tasks.

In the slips-of-action task, aside from the balance parameter DSI, no separate parameters describe the two individual instrumental systems separately. However, the DSI is calculated based on percentage responses to still valuable outcomes and the percentage slips of actions (i.e., responses for devalued outcomes). A higher amount of slips-of-actions to devalued outcomes is thought to resemble higher S-R habit responding. Therefore, we *post hoc* explored the direction of the association between the two systems by taking the two individual variables of the slips-of-action phase as a rough approximation of goal-directed and habitual behavior, and the MB and MF parameters of the two-step task.

As we had *a priori* hypotheses of a positive association between goal-directed and MB measures from the two tasks (Doll et al., 2012; Friedel et al., 2014; Gillan et al., 2015), we report one-tailed *p*-values. Additionally, we explored a positive association between habitual and MF behavior in the two tasks. Due to the non-normal distribution of the slips-of-action parameters and *β*_MF_, we applied the more conservative Spearman correlation coefficient, in line with a previous cross-validation study (Friedel et al., 2014).

## Results

For sample description and scores on the general cognitive tests, see Table 1.

### Three-Phase Instrumental Learning Task

As all variables of the instrumental learning task violated the assumption of normality (Shapiro-Wilk test: *p*’s < 0.05), we report median and interquartile range (IQR) in addition to the average percentages and used non-parametric tests where necessary.

#### Discrimination Training Phase

**Figure 3:**
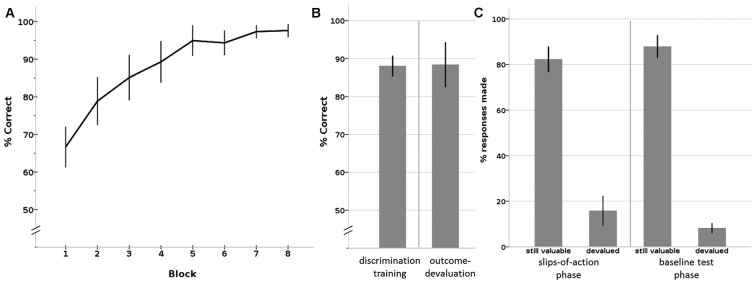
**Results of the Instrumental learning task. (A)** Instrumental discrimination training phase, displayed as learning over eight blocks. By the eighth block, all participants had learned the correct S-R-O contingencies significantly above chance level. **(B)** Average percentage correct responses on the (total) discrimination training phase (left) and outcome devaluation phase (right). **(C)** Percentage responses to still valuable and devalued trials of the slips-of-action phase (left) and the baseline test phase (right). Error bars: 95% confidence interval.

All participants showed the expected learning effect, confirmed by a Friedman test that showed a significant increase in percentage of correct responses over the eight blocks (*X*^2^ = 103.922, *p* < 0.001; see Figure 3A). A non-parametric binomial test shows that by the last block everyone had learned the correct responses to the stimuli significantly above chance level (*P*(*Y* > 50 | *n* = 28, *p* = 0.5) < 0.001), with an average percentage correct responses of 97.6% (SD = 4.45; median = 100%; IQR = 6.25) by block 8.

#### Outcome-Devaluation Test Phase

During the outcome test phase, participants showed an average percentage correct responses of 88.4% (SD = 15.17; median = 94.44; IQR = 10.42), which was also significantly above chance level (*P*(*Y* > 50 | *n* = 28, *p* = 0.5) < 0.001; see Figure 3B).

#### Slips-of-Action Phase

The crucial test of this task is the slips of action phase, where competition between outcome-based and stimulus-driven control is tested. Participants responded on average 82.2% (SD = 14.54; median = 85.42; IQR = 19.79) on stimuli that led to still-valuable outcomes. Slips of actions, that is, responding to stimuli that had a devalued outcome, occurred on average in 15.8% (SD = 16.92; median = 9.72; IQR = 13.19) of the devalued trials (see Figure 3C, left panel). The calculated DSI was 66.47 (SD = 29.60; median = 75.00; range: 26–95; IQR = 27.08), on average. Three statistical outliers (*z* > 2) had a DSI of around zero or below, mainly due to a low response rate on the stimuli associated with a still valuable outcome. These three participants also showed a deviating response pattern in the other phases (including discrimination training, outcome devaluation, baseline test) compared to the rest of the sample, by *z*-scores of or above |2|. For instance, they showed a response pattern of around chance level on the outcome devaluation phase and/or baseline test. As these three participants clearly did not show task participation, per chance by lack of attention or incomprehension of the instructions, we removed them from further correlation analyses.

The DSI showed a moderately positive correlation with other cognitive measures such as general intelligence, as assessed by the WMT and visual short-term memory, as assessed by the VPA (WMT: *ρ*_(25)_ = 0.356, *R*^2^ = 0.180, *p* = 0.040; VPA: *ρ*_(25)_ = 0.434, *R*^2^ = 0.400, *p* = 0.015). Cognitive processing speed, as measured with the DSST did not show a clear association with devaluation sensitivity (*ρ*_(25)_ = 0.274, *R*^2^ = 0.050, *p* = 0.108).

#### Baseline Test Phase

During the baseline test, participants responded on average to 87.8% of the still-valuable stimuli (SD = 12.81; median = 92.36; IQR = 11.46) and on average to only 8.2% (SD = 5.63; median = 6.94; IQR = 7.64) of the devalued stimuli (see Figure 3C, right panel). The difference between %responses to still valuable and devalued trials was 79.61 (SD = 14.11; median = 83.33; IQR = 14.58), on average. This difference score is significantly higher than the difference score (DSI) on the slips-of-action phase (see above; Wilcoxon Signed Rank Test: *Z* = −3.019, *p* = 0.002). This shows that participants had no problems inhibiting their responses after *stimulus devaluation*.

### Two-Step Sequential Decision-Making Task

**Figure 4:**
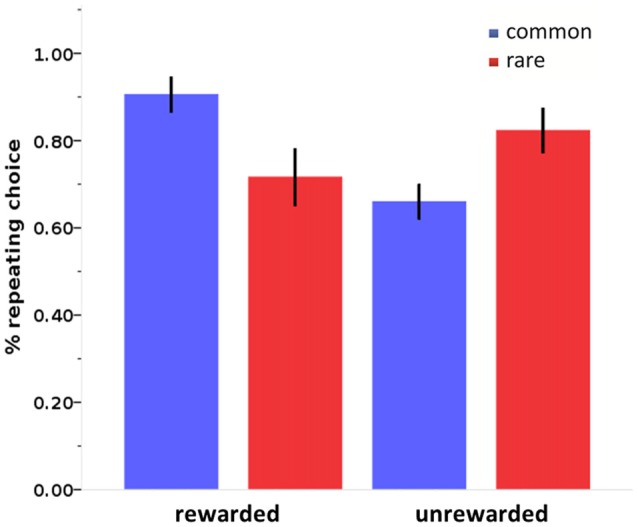
**Stay probabilities in the two-step sequential decision-making task.** Stay probabilities in the two-step sequential decision-making task show a reward by state interaction. Error bars: 95% confidence interval.

Stay probabilities showed a significant main effect of reward (*F*_(1,27)_ = 6.79, *p* = 0.015), as well as a reward by state interaction (*F*_(1,27)_ = 43.76, *p* < 0.001), but no main effect of state (*F*_(1,27)_ = 0.24, *p* = 0.628; see Figure 4). This result replicates previous studies with the same task (Daw et al., 2011; Wunderlich et al., 2012b; Smittenaar et al., 2013; Deserno et al., 2015a; Gillan et al., 2015) and reflects an influence of both rewards and stay transitions on choice behavior. We further quantified this with computational modeling using a hybrid RL model that weights the relative influence of the MF and MB strategies. Distribution of modeling parameters is displayed in Table 2.

**Table 2:** **Computational modeling parameter estimates**.

			Quantiles		
Parameter	Mean	SD	25%	50% (median)	75%
ω	0.68	0.07	0.62	0.7	0.73
β_MB_	5.28	1.80	3.64	5.58	6.51
β_MF_	2.39	0.89	1.67	2.17	2.76
β_2_	4.18	1.59	2.86	3.98	5.3
α_1_	0.50	0.15	0.39	0.51	0.64
α_2_	0.52	0.25	0.33	0.58	0.70
λ	0.52	0.24	0.33	0.52	0.71
ρ	0.13	0.03	0.11	0.13	0.16
–LL	179.41	37.45	155.76	186.19	209.51

*Legend. ω: relative influence of model-free (MF) and model-based (MB) values. *β*: stochasticity of the choices for the first stage, under the MB system (*β*_MB_), under the MF system (*β*_MF_) and the second stage (β_2_). α: learning rate for first (α_1_) and second (α_2_) stage. λ: reinforcement eligibility parameter (estimated value of the second stage should act as the same sort of MF reinforcer for the first stage choice). ρ: first-stage choice perseveration. –LL: negative log likelihood, indicating relative model-fit*.

The parameters of interest of the two-step task also showed a moderate association with other cognitive measures. The balance parameter ω only showed a significantly positive correlation with the VPA and a moderate but non-significant (trendwise) association with WMT and DSST (VPA: *ρ*_(25)_ = 0.422, *R*^2^ = 0.131, *p* = 0.018; WMT: *ρ*_(25)_ = 0.271, *R*^2^ = 0.058, *p* = 0.095; DSST: *ρ*_(25)_ = 0.301, *R*^2^ = 0.094, *p* = 0.087). The MB parameter *β*_MB_ was only significantly associated with the WMT but not (only trendwise) with the VPA (WMT: *ρ*_(25)_ = 0.388, *R*^2^ = 0.152, *p* = 0.028; VPA: *ρ*_(25)_ = 0.306, *R*^2^ = 0.086, *p* = 0.068; DSST: *ρ*_(25)_ = 0.070, *R*^2^ = 0.0036, *p* = 0.378), whereas the MF parameter *β*_MF_ was not correlated with any of the other cognitive measures (WMT: *ρ*_(25)_ = −0.010, *R*^2^ = 0.028, *p* = 0.482; VPA: *ρ*_(25)_ = −0.142, *R*^2^ = 024, *p* = 0.250; DSST: *ρ*_(25)_ = −0.212, *R*^2^ = 0.181, *p* = 0.172).

### Construct Validity: Correlation Between the Two Paradigms

**Figure 5:**
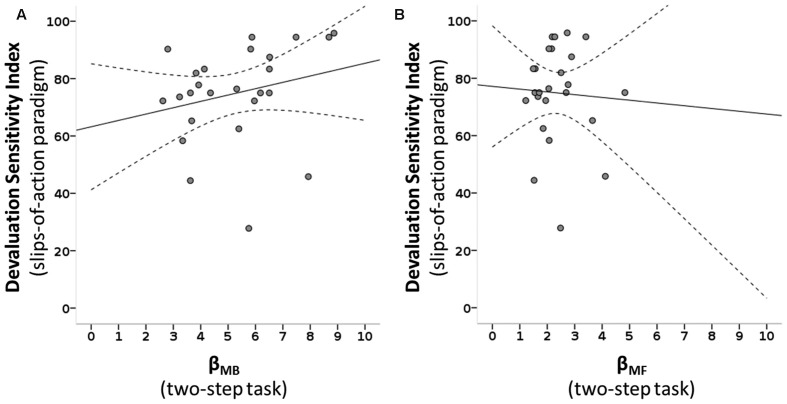
**Scatterplots of MB and MF choice values of the two-step task and devaluation sensitivity of the slips-of-action paradigm. (A)** A positive correlation is seen between the MB parameter of the two-step task (*β*_MB_) and the devaluation sensitivity index (DSI) of the slips-of-action task: *ρ*_(25)_ = 0.431, *R*^2^ = 0.055, *p* = 0.016. **(B)** No significant association is seen between the MF parameter of the two-step task (*β*_MF_) and the DSI: *ρ*_(25)_ = 0.172, *R*^2^ = 0.003, *p* = 0.205. Dotted lines: 95% confidence interval.

We tested how the parameters of the two-step task describing the individual influence of the two systems on choice behavior were related to devaluation sensitivity. The slips-of-action phase DSI correlated positively with *β*_MB_ of the two-step task (*ρ*_(25)_ = 0.431, *R*^2^ = 0.055, *p* = 0.016), surviving Bonferroni correction for the three correlations of interest that were performed, but not with *β*_MF_ (*ρ*_(25)_ = 0.172, *R*^2^ = 0.003, *p* = 0.205; see Figure 5). Next, we tested if a found association between the DSI and individual system parameters of the two-step was reflected in the balance parameter of the two-step task. We see a positive, albeit non-significant, relation between the balance parameters of the two tasks: ω of the two-step task and DSI of the slips-of-action phase correlated positively however, this was non-significant, but showed only a trend (*ρ*_(25)_ = 0.285, *R*^2^ = 0.035, *p* = 0.083).

Following the significant association between devaluation sensitivity and MB control we *post hoc* explored which of the two variables in the slips-of-action phase that contribute to the DSI score (% responses to valued and devalued trials), drove this significant association between *β*_MB_ and the DSI. The % responses on still valuable trials was positively associated with the MB variable (*ρ*_(25)_ = 0.477, *R*^2^ = 0.091, *p* = 0.008), whereas % responses to devalued trials (slips-of-action) was not (*ρ*_(25)_ = −0.123, *R*^2^ = 0.012, *p* = 0.279).

As goal-directed behavior in both tasks consistently correlated with an independent measure of visual short-term memory (VPA), we performed a *post hoc* mediation analysis (PROCESS Macro; Hayes, 2013) with bias corrected bootstrap confidence intervals to further elaborate a possible mediation factor in the three-way association. We entered VPA score as a mediator (M) in models with the DSI of the slips-of-action task as outcome variable (*Y*), and the MB parameter (*β*_MB_) of the two-step task as independent variable (*X*). This model was significant (*R*^2^ = 0.373, *F*_(1,23)_ = 6.556, *p* = 0.006), whereas the inverse model with *X* = DSI and *Y* = *β*_MB_ was not (*p* = 0.137). Interestingly, this suggests a direction, where MB learning (*β*_MB_) is a predictor for devaluation sensitivity, and not vice-versa. The direct effect between *X* and *Y* seemed to decrease when entering the mediators in the model (c’-path: *p* = 0.730). We tested the change from c to c’ with the conservative Sobel’s test, showing a moderate effect size, but no significance (c-c’: *k*^2^ = 0.186, *Z* = 1.301, *p* = 0.193, 95% CI = [0.03–0.44]). Note that although the Sobel test result is not significant per the *p*-value, the confidence interval does not include zero, which would lend support to the interpretation that there is a moderate effect size. Therefore, this mediation analysis points toward a partial mediation of visual short-term memory on the association between devaluation sensitivity and the individual MB parameter *β*_MB_.

## Discussion

The aim of the current study was to cross-validate instrumental behavior from the goal-directed and habit systems assessed by two frequently used tasks. To this end, we correlated parameters assessing the involvement of the two systems from an instrumental learning task with an instructed devaluation slips-of-action phase (de Wit et al., 2007, 2012b) and a two-step sequential decision-making task (Daw et al., 2011). Partly conforming to our hypothesis, we see that MB control in the two-step task is moderately associated with goal-directed behavior in the slips-of-action paradigm, as *β*_MB_ correlated with the DSI. This effect of the DSI seemed mainly driven by responding to still valuable trials, and not by responding to devalued trials. An association between MB control and devaluation sensitivity was also partly captured by a moderate, albeit only trendwise significant, correlation between DSI and the balance parameter of the two-step task (ω), which assesses a relative involvement of the MB and MF systems in choice behavior. MF control did not seem to be significantly associated with devaluation sensitivity, which could have attenuated the association between the two balance parameters including ω of the two-step task. Ergo, we find a very moderate cross-validation between these tasks on the assessment of behavior within the goal-directed system, whereas behavior within habit-like systems did not seem overtly related between the tasks.

A moderate correlation between MB learning and goal-directed devaluation sensitivity seems in agreement with a common framework to describe goal-directed behavior, as suggested by Dolan and Dayan (2013) and would indeed support common definitions of (aspects of) goal-directed behavior between the different task operationalizations. Comparably, in a previous construct validity study, Friedel et al. (2014) also found MB and goal-directed behavioral control to be positively correlated between the two-step sequential decision-making task (Daw et al., 2011) and a selective devaluation paradigm (Valentin et al., 2007), respectively. Furthermore, MB learning has been positively associated with goal-directed responding within one paradigm (Gillan et al., 2015). This is in line with the results in the current study, where we associated MB control with devaluation sensitivity between two separate paradigms. This suggests that computational accounts of MB control mirror, at least partly, one of the many aspects of (established) goal-directed behavior as measured with a selective devaluation paradigm.

However, we do have to caution that the amount of shared variance between both tasks was rather low. Moreover, it seems that MB behavior predicts performance on valued trials rather than responses towards devalued items, as it was mainly responding to still valuable trials on the slips-of-action phase that drove the association between devaluation sensitivity and MB behavior. Together, this suggests that each task does pick up distinct additional aspects of goal-directed behavior. It could be conceivable that the DSI more captures sensitivity to outcome value, whereas the two-step task (additionally) seizes sensitivity to outcome contingency; both part of the definition of goal-directed behavior. These distinct aspects of goal-directed behavior may be differently processed in the brain (however, for a study in primates, see Izquierdo et al., 2004). Nonetheless, considering the association between MB behavior and responses to still valuable items specifically, a part of the variance that is shared between the two constructs in this study might also be driven by performance more in general.

Interestingly, and in this line, both goal-directed and MB parameters were positively related to higher-order cognitive measures including visual short-term memory. Goal-directed behavioral control has been repeatedly shown to rely on higher-order cognitive measures, an effect most pronounced with working-memory capacity (Eppinger et al., 2013; Otto et al., 2013; Schad et al., 2014; Culbreth et al., 2016). Working memory capacity has even been shown to influence effects of detrimental environmental factors, such as stress on MB control (Otto et al., 2013). Although we did not directly measure working-memory capacity, we did have information on neurocognitive capacities in other cognitive domains. An exploratory mediation analysis indicated that short-term memory partly mediated the correlation between goal-directed and MB behavior in the two tasks, indicating that the tendency to be MB/goal-directed in each of the tasks depends on higher-order cognitive capacities, which could be part of the explanation of a moderate overlap between the two constructs.

Although negative results should be interpreted carefully, we would like to comment on the complete absence of a significant association between MF learning and habitual behavior in the two tasks, even when including the three participants that were regarded outliers on task behavior. We would like to discuss three possible explanations: (1) the assessments of the habitual system in the two tasks are unrelated; (2) habitual responding is the predominant mode of control leading to little variability, and thus correlation between paradigms; and (3) alternatively, the explanation might not lie at the level of construct, but in the (very goal-directed) sample tested. We discuss these explanations more in detail below.

The first intuitive explanation is that the differently measured aspects of habitual behavior are unrelated, either at the level of the two used paradigms, or more general at the level of construct definition. It is possible that the degree of MF control is not directly related to the propensity to form habits, but that the formation of action sequences might explain habitual actions, as suggested before Dezfouli and Balleine (2013). Contributing to this might be the distinction between assessing ongoing updating processes during the two-step vs. amount of slips of action as the expression of previously learned S-R associations. This might specifically be crucial for the assessment of the habit system. The acquisition vs. expression of habitual control is thought to be represented in distinct neural systems (Liljeholm et al., 2015; although integrative views have also been proposed), advancing the belief that behavioral acquisition vs. expression of habits is distinctively assessed. The two-step task has changing reward contingencies throughout the task and measures an ongoing (MF) TD learning process without reaching an asymptote, forming MF habit-like behavior by repeating previously rewarded choices without considering the task structure. A habit is thought to represent an automatized end-point of learning, while TD learning is, although slowly, still sensitive to changes in the environment. Conversely, the slips-of-action task evaluates the degree to which habitual behavior is expressed during an extinction test probing previously deterministically learned S-R associations. Therefore, it is possible that expressed behavior during an ongoing (MF) learning process differs from behavior observed during testing of established S-R associations. In comparison, the study by Gillan et al. (2015) associated MB and MF control within the same task with an instructed devaluation test assessing goal-directed and habitual behavior. In line with our findings they found that the degree of MB learning was also associated with devaluation sensitivity after the learning phase. MF learning however, was not associated with devaluation sensitivity, comparable to our results.

Second, an absent association between the tasks on the assessment of the habitual system might also reflect the robustness of the habitual/MF system, forming a predominant default mode of response (Wood and Rünger, 2016). The variability in the balance between the two decision-making systems might be predominantly driven by variability in the MB system, thus allowing cross-validation between paradigms in the goal-directed system specifically, but not the habit system. The ubiquity of the goal-directed system (but not the habit system) has been acknowledged previously (Doll et al., 2012). However, it remains to be established how variability vs. stability in both systems constitutes a balance between goal-directed and habitual behavior (Lee et al., 2014). A default mode of response from the habit system would explain why an imbalance between the two systems in e.g., addiction seems to be driven by decreased goal-directed behavior as opposed to increased habitual responding (Sebold et al., 2014), although the opposite has also been described (Gillan and Robbins, 2014). Devaluation insensitivity after overtraining would then result from impaired goal-directed control instead of heightened habit formation. Of course, the strongest evidence for the notion that not either one, but a balance between two systems determines outcome devaluation sensitivity, comes from animal research, where a double dissociations is described: animals with lesions to dorsolateral striatum and infralimbic cortex are perpetually goal-directed, even after extensive training, whereas animals with dorsomedial striatal and prelimbic cortical lesions are habitual even after only minimal training (Yin et al., 2004, 2005b, 2006). It remains possible that the currently availably human tasks do not offer an adequate translation from the animal paradigms, and that the two tasks under scrutiny may not be optimally tailored to assess the contribution of habits in instrumental behavior. Importantly, neither of the paradigms compared in this study assesses full “end-stage” habits, which are typically manifest only after extensive overtraining (Colwill and Rescorla, 1988; Dickinson, 1994, 2015). Training in the current tasks lasts ten to 20 min, specifically the training phase of the instrumental learning task takes 16 encounters of every possible S-R-O association, which is more than the minimally needed amount to establish stable stimulus-based R-O associations, but less than some other studies (e.g., Tricomi et al., 2009). It therefore seems likely that these tasks fail to induce full end-stage habits. This complicates the discussion of habits for these tasks, and could further explain a lack of commonalities between the MF/habit constructs of the tasks. This end-stage phenomenon might not apply to the goal-directed system, as it is by definition more flexible and updated continuously, even after overtraining. The question further rises how these tasks under scrutiny relate to other tasks used to measure habit strength or related constructs, such as S-R instrumental learning tasks employing overtraining, skill learning tasks, spatial navigation tasks, the weather prediction task measuring implicit habit-like learning, and many others (Knowlton et al., 1994; Salmon and Butters, 1995; Gluck et al., 2002; Boettiger and D’Esposito, 2005; Marchette et al., 2011; Wood and Rünger, 2016).

As a third possibility for the absent association, it should be noted that the currently tested young and highly educated sample shows relatively dominant goal-directed (MB) behavior, which could (further) contribute to the fact that the current study only captures correlations between the tasks on the goal-directed systems. The average weighting parameter ω from the two-step task lies around 0.70, which is high compared to previous studies using the two-step task in healthy samples (Schad et al., 2014; Deserno et al., 2015a,b; Voon et al., 2015a,b; Morris et al., 2016; Worbe et al., 2016) and quantitatively indicates high involvement of the MB system. A predominant involvement of the MB system within this highly educated sample could lead to low variability or even a bottom-effect in the habitual system, rendering it harder to capture correlations between the MF/habit systems. Indeed, we see lower variability in the MF system than in the MB system, expressed by a lower variance of the *β*_MF_ parameter (see Table 2). Interestingly, and affirmatively, on the slips-of-action phase we see a relatively low percentage of responses to devalued trials (~15% slips-of-actions), compared with existing literature, where the percentage slips-of-actions in healthy samples on averages lies around ~30%–50% (Delorme et al., 2016; Ersche et al., 2016), indicating that in the current sample the habitual mode of control is relatively low in the slips-of-action phase. In line with the study by Gillan et al. (2015), this could be directly related to the relatively high involvement of the MB system during learning, as they have shown that MB learning protects against forming habits.

A limitation to this study is that due to the correlational associations between the tasks, we cannot elaborate on possible causal relationships between MB/MF learning and the expression of goal-directed/habitual responding during a devaluation test. The *post hoc* mediation analysis we performed did suggest directionality between MB control in the two-step task and devaluation sensitivity in the slips-of-action paradigm. This matches the directional association between MB control and devaluation sensitivity as reported by Gillan et al. (2015). However, in the current study set-up we can only refrain from further elucidations on this directionality.

In conclusion, the current study partly confirms a common framework between assessments of goal-directed and MB behavior, but we do not find such commonalities amongst the MF and habit system. However, the evidence is not strong: it should be acknowledged that the effects were only moderate, and the found associations explained only a small part of the shared variance, indicating there are still different aspects of goal-directed/MB behavior being picked up by the two tasks. Future studies should further elucidate these aspects, and the role of MF learning in forming habits. Moreover, systematic cross-validation on neural correlates of both instrumental decision-making systems is needed to further promote definition and adequate assessment of goal-directed and habitual behavior in healthy and diseased samples.

## Author Contributions

FS, AH and SW initiated the study. FS, AH, SW, AD and LD designed the study. AD and LD collected the data. LD and ZS performed the analyses. AD, LD, FS, AH and ZS interpreted the results. ZS drafted the article. ZS, AD, LD, SW, AV, H-JH, FS and AH read and corrected versions of the manuscript.

## Conflict of Interest Statement

The authors declare that the research was conducted in the absence of any commercial or financial relationships that could be construed as a potential conflict of interest.

## Abbreviations

ANOVA: Analysis of Variance
DSI: Devaluation Sensitivity Index
DSST: Digit Symbol Substitution Test
EEG: Electro Encephalography
IQR: Interquartile Range
ITI: Inter-trial Interval
MB: Model-based
MF: Model-free
ms: Milliseconds
O: Outcome
Q: Choice Values
R: Response
RL: Reinforcement Learning
S: Stimulus
SARSA: State-Act-Reward-State-Act
SD: Standard Deviation
TD: Temporal Difference
TMT: Trail Making Test
VPA: Visual Paired Association Test
WMT: Wiener Matrizen Test.
